# Supplementary material for: DIRT/3D: 3D root phenotyping for field-grown maize (Zea mays)
Source: Plant Physiol. 2021 Jul 8;187(2):739–57. doi: 10.1093/plphys/kiab311 (PMC8491025; doi:10.1093/plphys/kiab311)
Supplement: kiab311_Supplementary_Data [file kiab311_supplementary_data.zip › supplemental data SD1.docx]

### **DIRT/3D models**

**DOI:**

10.25739/vf8r-z736

**Permanent link:**

<https://datacommons.cyverse.org/browse/iplant/home/shared/commons_repo/curated/Bucksch_DIRT3D-Models-PlantPhysiology_Jun2021>

**Creator:**

Alexander Bucksch

**Description:**

3D point cloud data of maize roots

**Publisher:**

Plant Physiology, CyVerse Data Commons

**Publication Year:**

2021

**Rights:**

This data is made available under the Public Domain Dedication and License v1.0 whose full text can be found at <http://www.opendatacommons.org/licenses/pddl/1.0/>

**Citation:**

Alexander Bucksch (2021). DIRT/3D models. Plant Physiology, CyVerse Data Commons. DOI 10.25739/vf8r-z736
