# Supplementary material for: DIRT/3D: 3D root phenotyping for field-grown maize (Zea mays)
Source: Plant Physiol. 2021 Jul 8;187(2):739–57. doi: 10.1093/plphys/kiab311 (PMC8491025; doi:10.1093/plphys/kiab311)
Supplement: kiab311_Supplementary_Data [file kiab311_supplementary_data.zip › supplemental data SD3.docx]

**Source code is available on GitHub:**

<https://github.com/Computational-Plant-Science/3D_model_reconstruction_demo>

<https://github.com/Computational-Plant-Science/3D_model_traits_demo>

**Preconfigured containers are available on Docker Hub:**

<https://hub.docker.com/r/computationalplantscience/3d-model-reconstruction>

<https://hub.docker.com/r/computationalplantscience/3d-model-traits>
