# Supplementary material for: DIRT/3D: 3D root phenotyping for field-grown maize (Zea mays)
Source: Plant Physiol. 2021 Jul 8;187(2):739–57. doi: 10.1093/plphys/kiab311 (PMC8491025; doi:10.1093/plphys/kiab311)
Supplement: kiab311_Supplementary_Data [file kiab311_supplementary_data.zip › supplemental figures.pdf]

Supplemental Figure 1: Flow diagram of the DIRT3D software pipeline

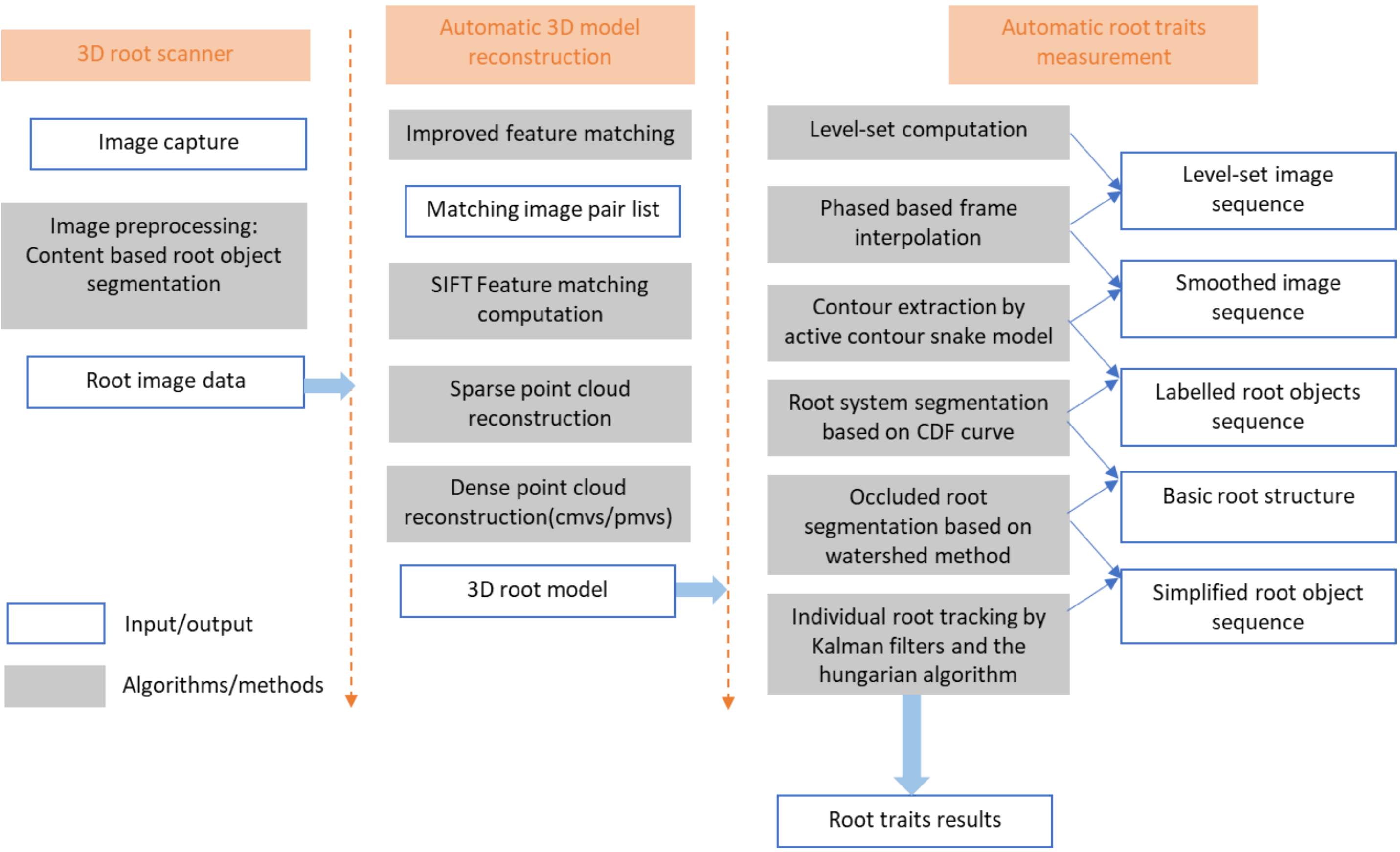

Supplemental Figure 2: Design plans of the 3D root scanner

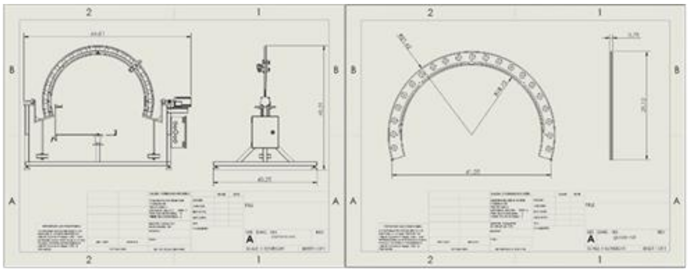

(a) Mechanical parts design

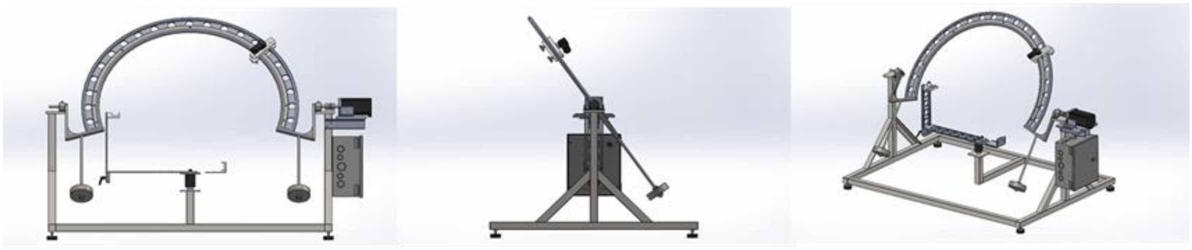

(b) Projection of frontal plane, profile plane and oblique plane

Supplemental Figure 3: Smoothing of level-set images to improve tracking of individual roots

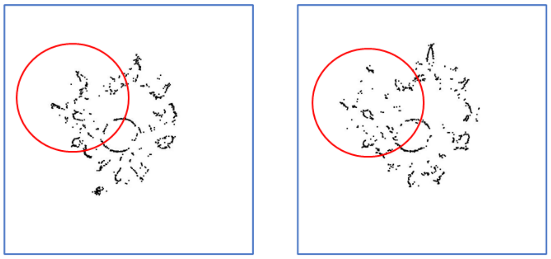

(a) Two consecutive level set images

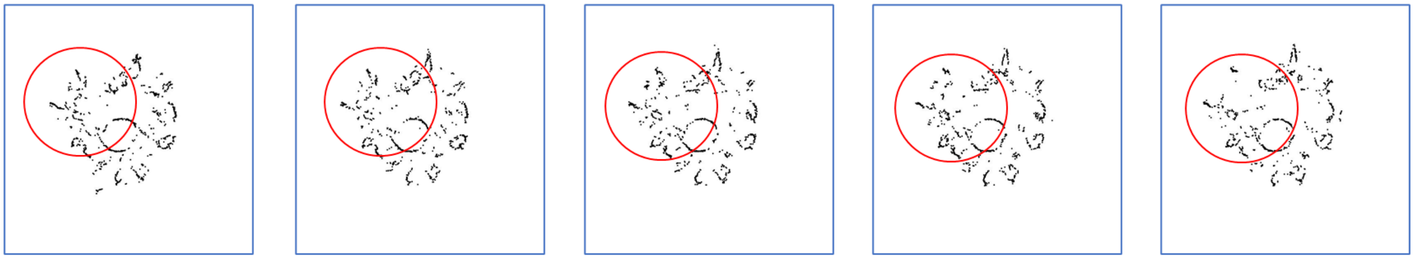

(b) Consecutive level set images after phase-based frame interpolation

Supplemental Figure 4: Detecting roots with active contour snakes

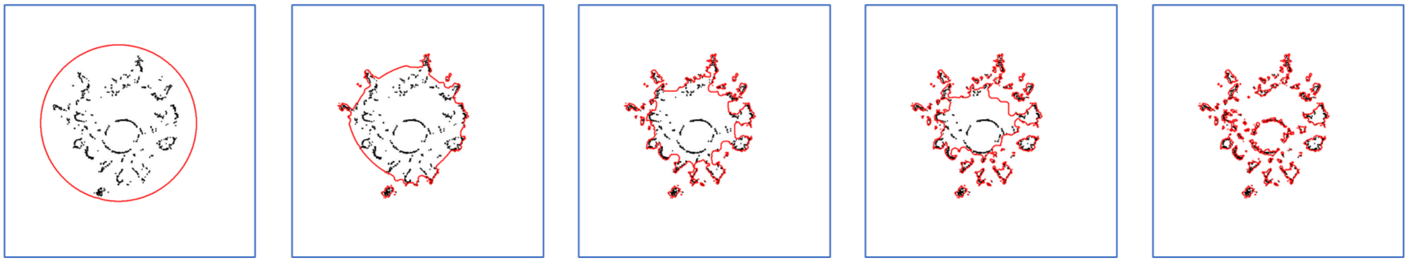

Supplemental Figure 5: Watershed segmentation to resolve occlusion effects

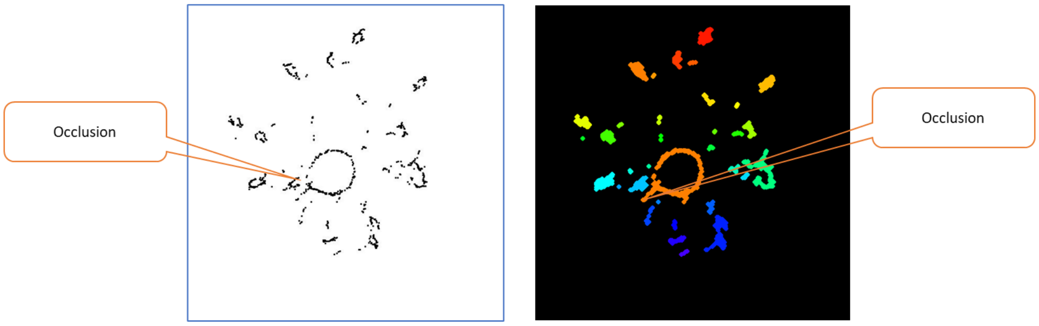

(a) sample level set image

(b) Active snake result of (a)

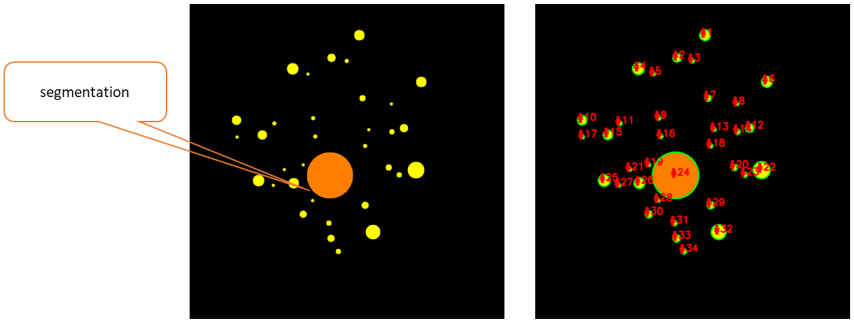

(c) Watershed Segmentation result of (b)

(d) Number labelling result of (c)

Supplemental Figure 6: Validation of 10 traits against manual ground truth

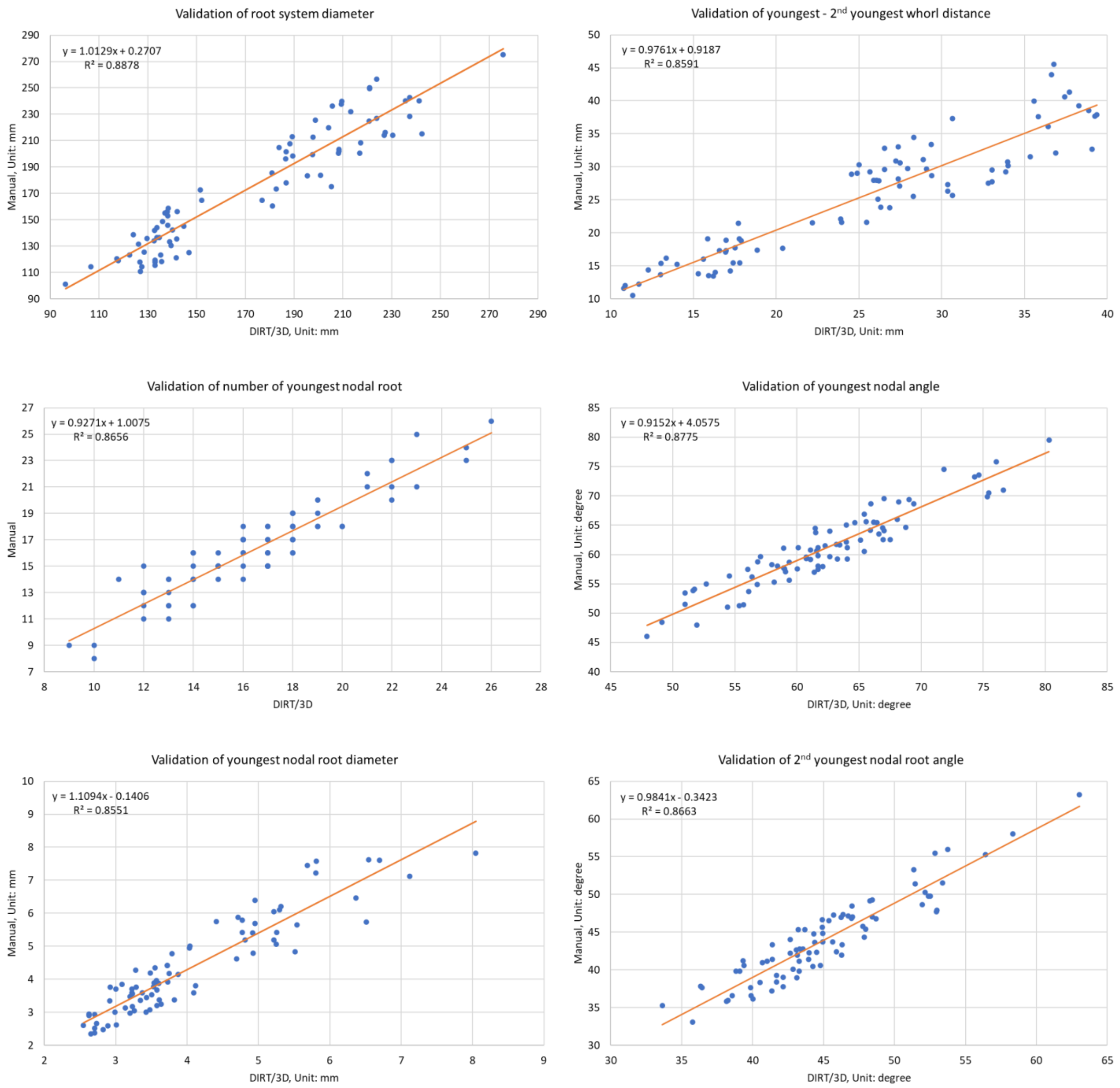

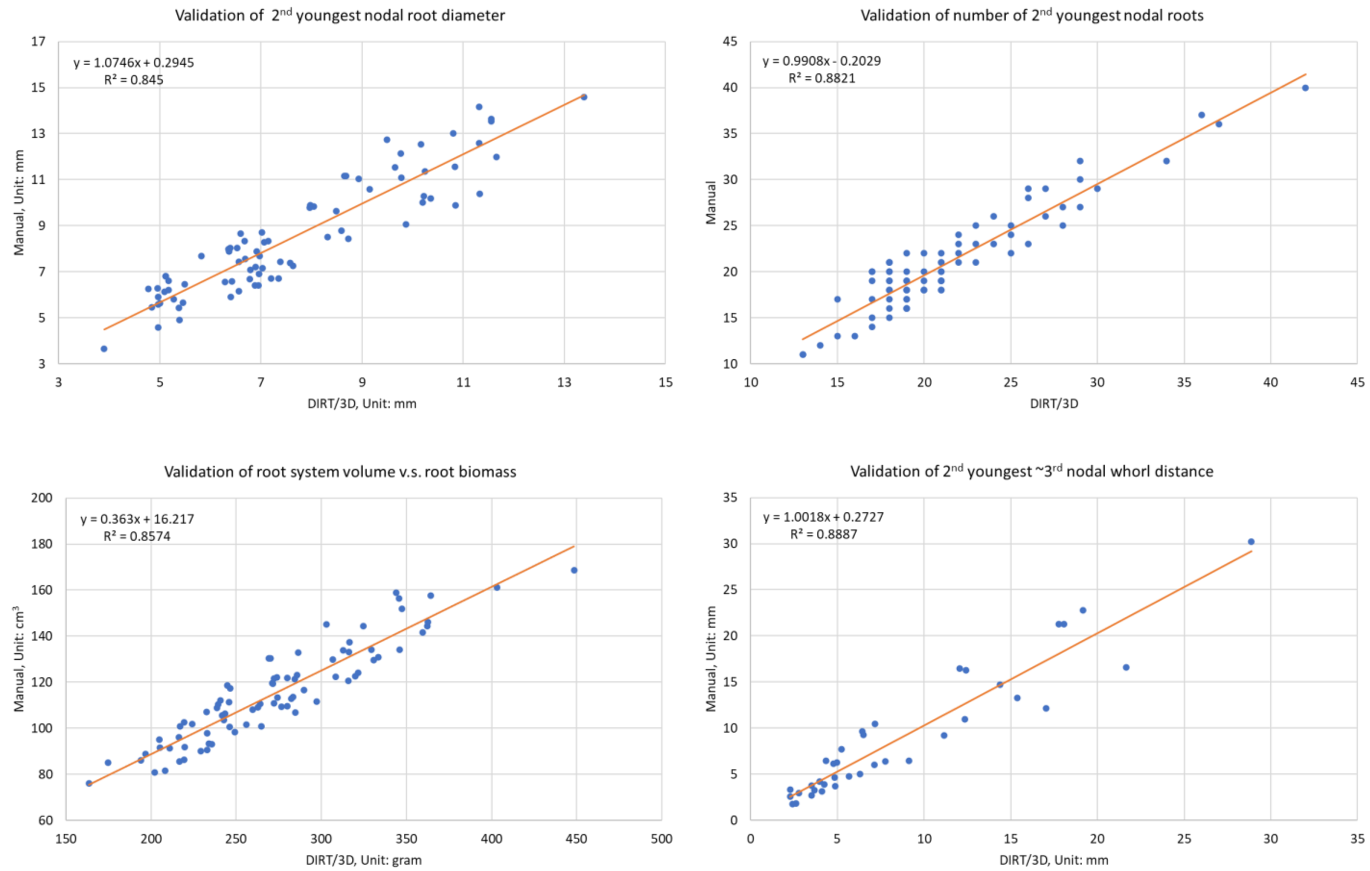

Supplemental Figure 7: Principle of whole root descriptor

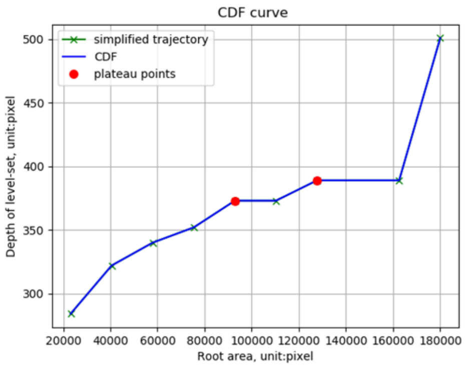

(a) Example of a cumulative distribution function to calculate the whole root descriptor.

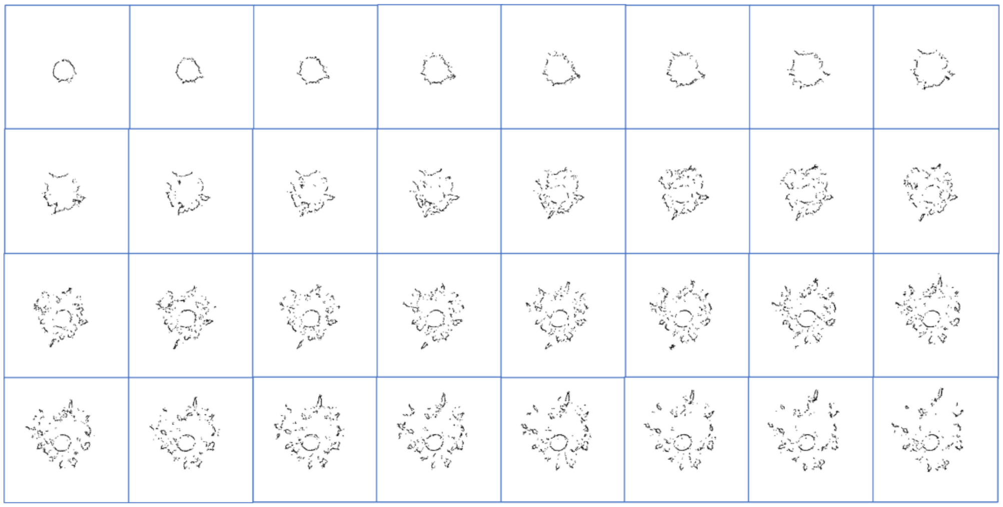

(b) Consecutive sample images selected from generated level set image sequence.

Supplemental Figure 8: Design of the Raspberry Pi cluster in the 3D root scanner

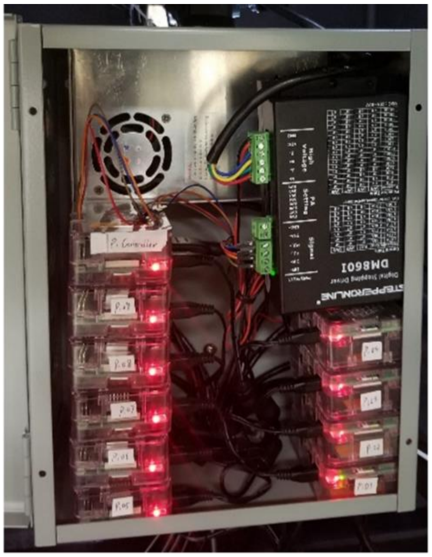

(a) Raspberry Pi 3 cluster and stepper motor drive unit

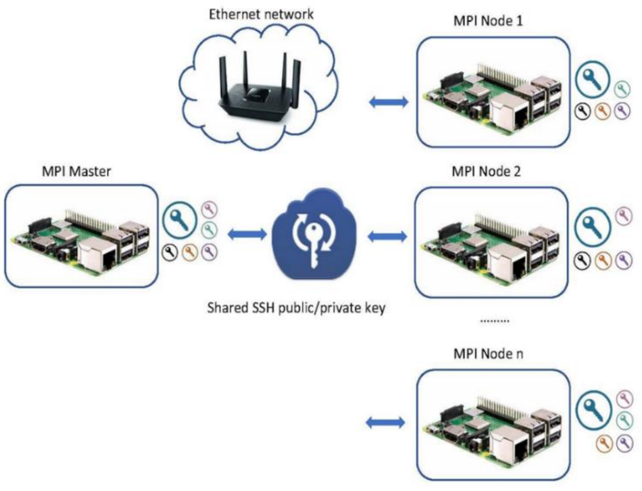

(b) Interface configuration for passing messages between server and client

Supplemental Figure 9: Example of a blurred image

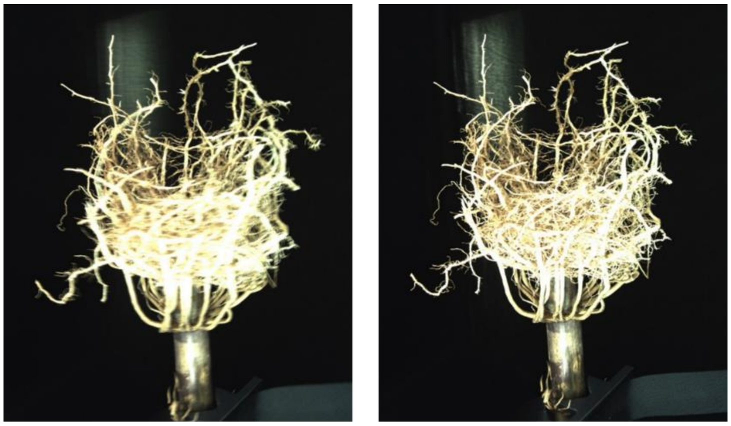

Comparison of a blurred and sharp image obtained with the 3D root scanner

Supplemental Figure 10: Contend based root object segmentation

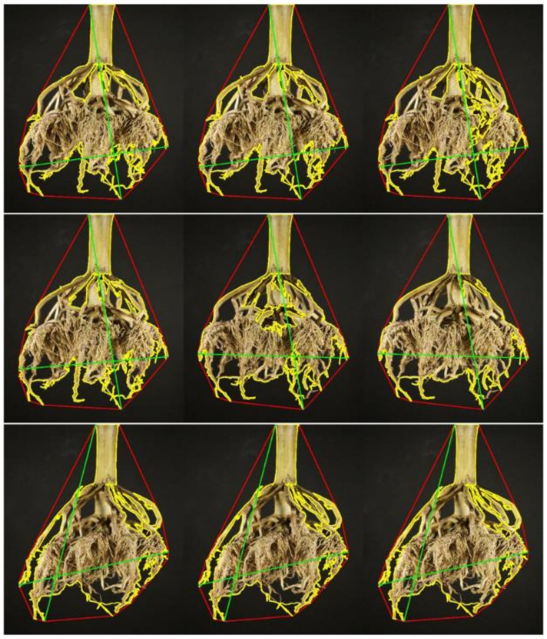

Supplemental Figure 11: False feature matchings

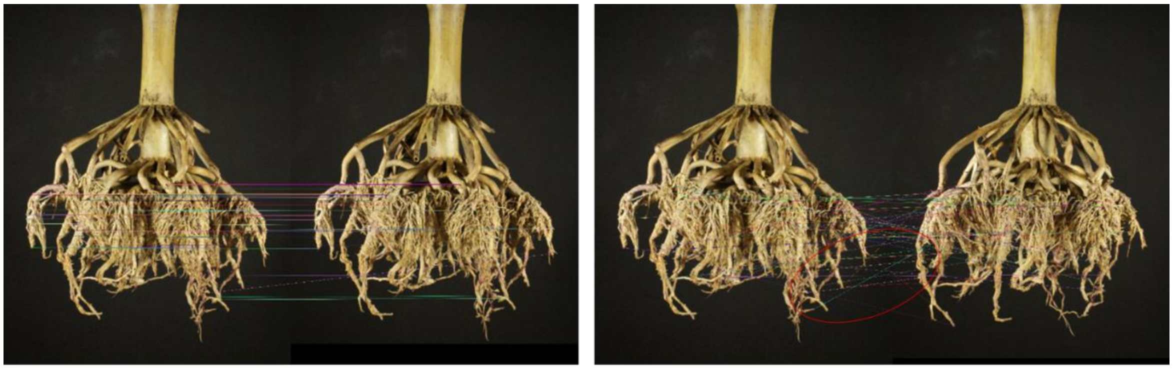

(a) Adjacent image pair

(b) Distant image pair

Supplemental Figure 12: Principle of the sliding window

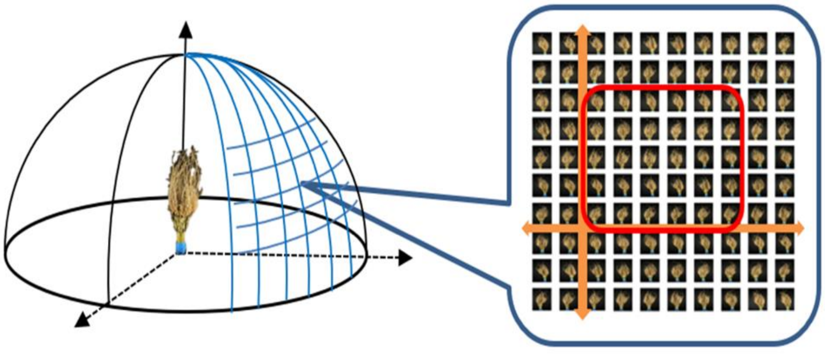

(a) Imaging set up

(b) Sliding window

Supplemental Figure 13: A top-down level set scan of the 3D root model to compute 3D root traits

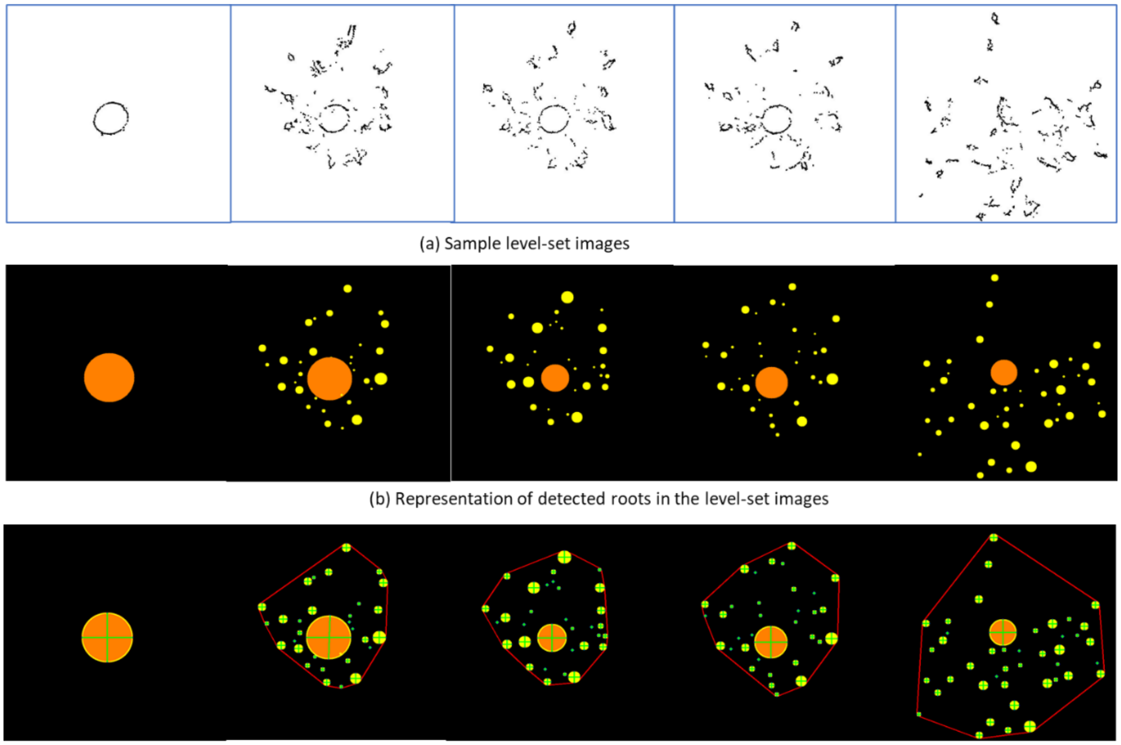

(a) Sample level-set images

(b) Representation of detected roots in the level-set images

(c) Computation of individual and system level root traits
